# Retinal Structure in *RPE65*-Associated Retinal Dystrophy

**DOI:** 10.1167/iovs.61.4.47

**Published:** 2020-04-29

**Authors:** Neruban Kumaran, Michalis Georgiou, James W. B. Bainbridge, Mette Bertelsen, Michael Larsen, Fiona Blanco-Kelly, Carmen Ayuso, Hoai Viet Tran, Francis L. Munier, Angelos Kalitzeos, Michel Michaelides

**Affiliations:** 1 UCL Institute of Ophthalmology, University College London, London, United Kingdom; 2 Moorfields Eye Hospital NHS Foundation Trust, London, United Kingdom; 3 Guy's and St. Thomas’ NHS Foundation Trust, London, United Kingdom; 4 Rigshospitalet, Copenhagen, Denmark; 5 University of Copenhagen, Copenhagen, Denmark; 6 Fundación Jiménez Díaz University Hospital, (IIS-FJD, UAM), Madrid, Spain; 7 Centro de Investigacion Biomedica en Red de Enfermedades Raras (CIBERER), ISCIII, Madrid, Spain; 8 Jules-Gonin Eye Hospital, Fondation Asile des Aveugles, Université de Lausanne, Lausanne, Switzerland

**Keywords:** retinal structure, optical coherence tomography (OCT), autofluorescence, FAF, retina, leber congenital amaurosis, RPE65, LCA, LCA2, clinical trials, endpoints, natural history

## Abstract

**Purpose:**

*RPE65*-associated retinal dystrophy (*RPE65*-RD) is an early onset, progressive, severe retinal dystrophy. We sought to characterize the natural history of retinal degeneration in affected individuals.

**Methods:**

We performed cross-sectional and longitudinal quantitative and qualitative assessments of retinal architecture in *RPE65*-RD using spectral domain optical coherence tomography (SD-OCT) and fundus autofluorescence (FAF) imaging. Twenty-six subjects (mean age, 14.8 years, range, 5–24 years) with *RPE65*-RD underwent SD-OCT and FAF imaging, of whom 14 subjects were followed up over time. Foveal thickness (FT), outer nuclear layer thickness (ONLT), ellipsoid zone width (EZW), and ellipsoid zone area (EZA) were calculated where possible. These were correlated with age, best corrected visual acuity (BCVA), and central 30° retinal sensitivity (V_30_). Intra-observer agreement, test-retest repeatability, and interocular symmetry were also investigated.

**Results:**

We identified structural interocular symmetry, the presence of autofluorescence in 46% (12/26) of subjects, and the presence of foveal hypoplasia (associated with significantly worse BCVA) in 50% of subjects. EZW and EZA were measurable in 67% (35/52) and 37% (19/52) of eyes, respectively, with both demonstrating good agreement on repeated measurement. The annual rate of progression using EZW was −300.63 µm/year, and −1.17 mm^2^/year in EZA. EZW was found to have a statistically significant correlation with BCVA and V_30_.

**Conclusions:**

We identified the presence of autofluorescence in half of our subjects, with foveal hypoplasia also noted in half of our cohort. EZW, and to a lesser extent EZA, were robust measures of retinal degeneration and represent valuable metrics to determine the impact of intervention. (ClinicalTrials.gov number NCT02714816.)

Leber congenital amaurosis (LCA) is an inherited retinal disease that presents, in the majority, as a progressive rod-cone dystrophy.^1^ It affects between 1 in 33,000 and 1 in 81,000 live births and 25 genes to date have been identified as causing approximately 70% to 80% of cases. *RPE65*-associated retinal dystrophy (*RPE65*-RD) is thought to account for approximately 5% to 16% of LCA, and successful gene replacement has been demonstrated in phase I/II and III clinical trials.^2^^–^^10^ This has resulted in *RPE65*-RD becoming the first ocular condition for which an approved treatment is now available.

Several studies have investigated multiple aspects of retinal function in *RPE65*-RD, including identifying progressive loss of retinal sensitivity over the first three decades of life, residual color discrimination in adults, and reduced cone-driven temporal sensitivity.^11^^–^^15^ In contrast, retinal structure has only been investigated with optical coherence tomography (OCT). Initial time domain OCT (TD-OCT), has been superseded by spectral domain OCT (SD-OCT), which allows greater resolution, increased scanning speeds, and greater density scans, while minimizing eye motion artifacts.^16^ The first retinal lamination scan by means of TD-OCT in a subject with *RPE65*-RD identified an intact foveal contour, with qualitative thinning of the outer retina-choroid complex (albeit with limited resolution).^17^ Subsequent quantitative cross-sectional OCT studies have used a mixture of TD-OCT and SD-OCT. Overall, retinal thickness has been shown to be within normal limits, or evidence of central preservation surrounded by retinal thinning, with no clear relationship between age and retinal thickness.^18^ Following this, Jacobson et al*.* initially investigated outer nuclear layer (ONL) thickness in 11 children with *RPE65*-RD, and suggested that cone photoreceptors are partially lost early in childhood, but residual cones can persist for decades and show a slow age-related decline.^19^ Nine children (including seven of the above) were further imaged, and it was suggested that the ONL was, on average, thinner inferiorly, in comparison to relative preservation of ONL in the superior-temporal and temporal pericentral retina.^20^ Through further expansion of the above 2 cohorts to 20 subjects (adults and children), the same group showed evidence that dark-adapted retinal sensitivity may be associated with ONL thickness.^21^ Most recently, Chung et al. undertook a retrospective analysis of OCT scans in 32 subjects with *RPE65*-RD, investigating only retinal thickness and ONL thickness, demonstrating no effect of age on either measure.^22^

Greater consensus and understanding of anatomic landmarks using SD-OCT now exists, with the hyper-reflective zone, previously thought to correlate with the inner and outer photoreceptor segments, now proposed to be the ellipsoid component of the photoreceptors and termed the ellipsoid zone (EZ).^23^ Subsequently, many groups have investigated both the EZ width (EZW) and the EZ area (EZA), and characterized its applicability, measurement, and repeatability in conditions such as retinitis pigmentosa, with these metrics now accepted by the US Food and Drug Authority (FDA) and European Medicines Agency (EMA) as valid clinical trial outcome measures.^24^^–^^27^ However, to date, EZW and EZA analysis has not been performed on subjects with *RPE65*-RD.

Fundus autofluorescence (FAF) is a noninvasive imaging modality that utilizes the autofluorescent properties of lipofuscin and other fluorophores. Increased lipofuscin levels have been correlated with photoreceptor loss.^28^ Initially, three subjects with LCA were noted to have a variety of FAF phenotypes, with a normal, increased, and decreased autofluorescence described.^29^ However, following a dedicated study in 10 subjects with *RPE65*-RD, it was suggested that *RPE65*-RD is associated with reduced or absent autofluorescence.^30^ In direct contrast, increased autofluorescence at the macula was noted in three of four *RPE65*-RD subjects with a hypomorphic phenotype and significantly better visual function.^31^ Further study of FAF is needed in a larger cohort of *RPE65*-RD to better describe the range and prevalence of the aforementioned FAF phenotypes.

Herein, we present cross-sectional and longitudinal data on retinal structure in a large cohort of patients with *RPE65*-RD using SD-OCT and FAF. We quantify total retinal and outer nuclear layer foveal thickness, EZW, and EZA, and also investigate measures of agreement, repeatability, and correlation with visual acuity and retinal sensitivity. Furthermore, we determine the prevalence of foveal hypoplasia and describe the range of FAF findings.

## Methods

### Subjects

A total of 26 subjects with molecularly confirmed *RPE65*-RD were assessed using SD-OCT and FAF imaging as part of a prospective natural history study (ClinicalTrials.gov number: NCT02714816). The study protocol adhered to the Tenets of the Declaration of Helsinki and was approved by the Moorfields Eye Hospital Ethics Committee. Informed consent was obtained from all adult subjects, whereas informed consent and assent were obtained from parents and children, respectively, prior to entering the study.

### Spectral Domain OCT

Subjects’ pupils were dilated using tropicamide 1% and phenylephrine 2.5%. Horizontal, high-resolution volume scans covering a 20° square area with 97 B-scans were acquired by dedicated research ophthalmic technicians, using the Heidelberg Spectralis OCT2 (Heidelberg Engineering, Heidelberg, Germany). Similarly, horizontal, transfoveal, high-resolution line (30°) scans were acquired. Automated real-time tracking (ART) with an average of at least 12 scans was used. For both volume and line scans, where nystagmus prevented acquisition with such settings, first high-resolution mode was replaced with the high-speed setting, and subsequently, if needed, ART was decreased. Both volume and line SD-OCT scans were automatically registered to a near infrared reflectance (NIR-R) fundus image. For the purposes of longitudinal analysis, the follow-up mode was used to ensure imaging of the same retinal region over time. Furthermore, all subjects underwent measurement of axial length using the Zeiss IOL master (Zeiss, Oberkochen, Germany).

**Figure 1.**
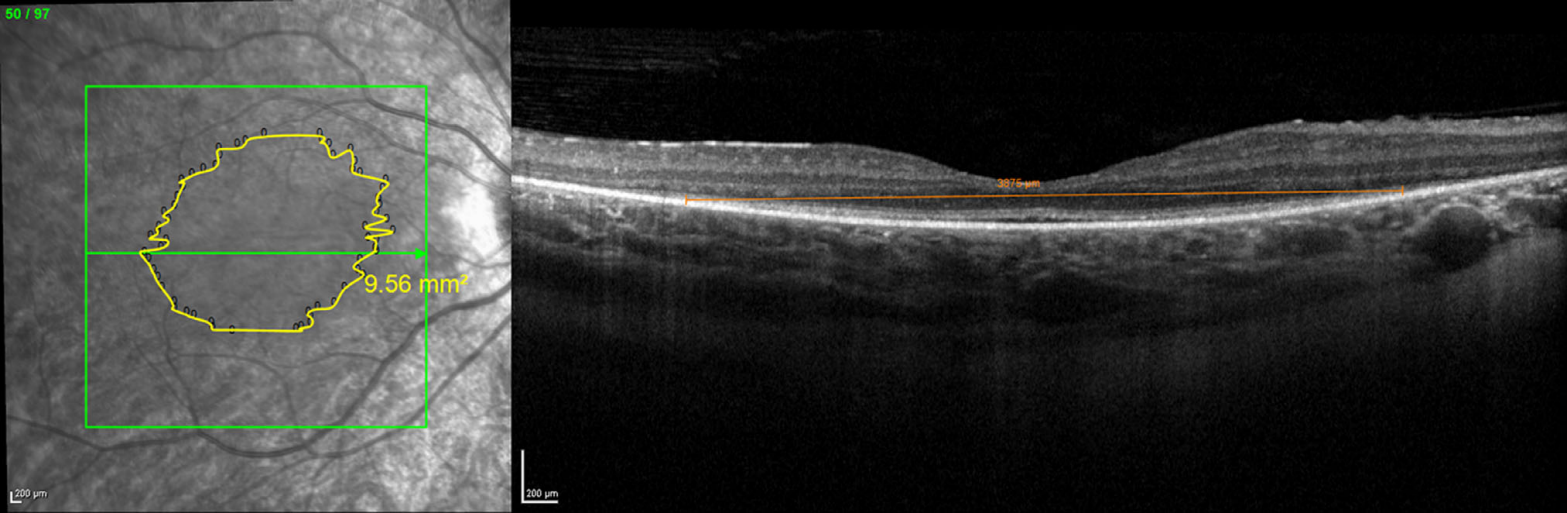
Example of SD-OCT en-face analysis. Example of the demarcated boundary on the infrared image of subject MM_0289, used to calculate the ellipsoid zone area (EZA), measured here as 9.56 mm^2^. Edges of the demarcated boundary shown on one B-scan using calipers as an example. The same caliper tool was used for ellipsoid zone width (EZW) measurements, but notably this was done using the 1 µm: 1 µm setting.

Vendor supplied Heidelberg Eye Explorer (Heyex) software version 1.6.1.0 was used for image analysis and quantification of EZW and EZA. The Heyex software caliper tool was used to measure EZW between the two edges of the B-scan where the hyper-reflective EZ converged with the proximal border of the retinal pigment epithelium.^32^ For EZA, each consecutive B-scan, within the volume of scans, was reviewed and the corresponding areas of EZ loss marked on the NIR-R fundus image. The area of the EZ loss was then calculated using the NIR-R fundus image with the region finder tool of the Heyex software.^33^ Figure 1 demonstrates an example of how EZA was measured. Heyex caliper measurements assume an axial length of 24 mm. To adjust for this, EZW measurements were multiplied by the subject's individual axial length (AL) divided by 24. Similarly, EZA measurements were multiplied by the individual AL divided by 24, to the power of 2, to account for quadratic scaling.

Foveal thickness (FT) and foveal ONL thickness (ONLT) were calculated from longitudinal reflectivity profiles (LRPs) generated from a five pixel sampling window, from the SD-OCT line scans, using ImageJ, as previously described.^34^^,^^35^ The ONL at the fovea was defined as the hyporeflective band between the inner limiting membrane (ILM) and external limiting membrane (ELM). Furthermore, at baseline assessment, both volume and line scans were reviewed for the presence or absence of foveal hypoplasia, defined as the persistence of one or more inner retinal layers (outer plexiform layer, inner nuclear layer, inner plexiform layer, or ganglion cell layer)^36^ through the fovea by one grader (N.K.). In ambiguous cases, consensus grading was established by two independent graders (N.K. and M.M.). In the presence of foveal hypoplasia, the ONL was defined as the hyporeflective band between the ELM and outer plexiform layer (OPL), as in previous studies.^37^^,^^38^

### Fundus Autofluorescence 

Fundus autofluorescence (FAF; short-wavelength) images were acquired and reviewed with the hardware and software described above. Both 30° and 55° square regions centered on the fovea were obtained. Images were taken with the intensity excitation light at 100% laser power, using ART with an average of 15 images. Images were graded qualitatively as having present or absent autofluorescence at baseline and follow-up visits by one grader (N.K.). Grading was performed in a blinded manner, with the grader unaware of the subject or previous grade, when monitored longitudinally.

### Visual Acuity

Best corrected LogMAR visual acuity (BCVA) was assessed, monocularly, with an Early Treatment Diabetic Retinopathy Study chart. Subjects were read standardized instructions.^39^ Precision Vision lightboxes were used (Precision Vision, Woodstock, IL, USA) and were illuminated with 2 cool daylight 20 watt fluorescent tubes, with the room lights turned off, so that no more than 161.4 lux should fall at the center of the chart. LogMAR values were calculated from the number of letters read, where the higher the LogMAR value, the worse the BCVA.

### Static Full-Field Perimetry

Static full-field perimetry was performed, monocularly, using the Octopus 900 (Haag Streit AG, Köniz, Switzerland) and EyeSuite software. The test was conducted monocularly in a dark room. Subjects were read standardized instructions^39^ and instructed to fixate on a cross with a background illuminance of 10 cd/m^2^ (31.4 apostilbs). Static perimetry used the German Adaptive Threshold Estimation (GATE) strategy (Haag Streit AG), stimulus size V, a 200 ms presentation, and a radially designed, centrally condensed grid of 164 test locations that extended radially to 80° temporally, 67° inferiorly, and 55.5° nasally and superiorly. Visual Field Modeling and Analysis software (VFMA, Office of Technology Transfer and Business Development [OHSU], Portland, OR, USA) was used to calculate the volume of the central 30 degrees of retinal sensitivity quantified in decibel-steradian (dB-sr), using previously reported methods.^13^^,^^40^^,^^41^

### Statistical Analysis

All statistical analysis was performed using the Stata software (StataCorp, College Station, TX, USA). In preparation for investigating interocular symmetry, baseline and rate of change data for FT, ONLT, EZW, and EZA were investigated for normality from left eyes and right eyes separately. Normality was assessed using the Shapiro-Wilk test, investigating skewness and reviewing the histograms. Subsequently, interocular symmetry of baseline measurements and the rate of change for all four metrics was investigated using a Wilcoxon matched-pairs signed-ranks test.

To minimize the clustering effect of using data from both eyes, for baseline assessments only, results from the left eyes of all subjects were analyzed. To assess agreement of EZW and EZA measurement, all baseline images were measured twice by a single observer (N.K.) a minimum of two weeks apart and in a blinded fashion. Agreement was investigated using the method popularized by Bland and Altman. Baseline FT, ONLT, EZW, and EZA were examined for normality using the Shapiro-Wilk test, calculating skewness and reviewing the associated histograms. All correlation data were investigated using the Pearson's product-moment correlation coefficient (r).

Longitudinal rate of change data for each of the three metrics (ONL, EZW, and EZA) is provided as a difference between the final measurement and baseline measurement, divided by the time between the two. Furthermore, a paired *t*-test was run to investigate if there was a difference between baseline and final follow-up measurements.

A binomial logistic regression was run to explore the effect of age on the presence (or absence) of increased autofluorescence on FAF imaging. Furthermore, an independent *t*-test was run to explore any potential relationships between BCVA and the presence or absence of foveal hypoplasia. Finally, the relationship between the presence of increased foveal thickness over time and the presence of foveal hypoplasia were investigated using a χ^2^ test of independence.

## Results

### Patient Demographics

Twenty-six subjects with molecularly confirmed *RPE65*-RD were enrolled (Table 1). Of the 26 patients, 13 were men. Ages ranged from 5 years to 24 years, with a mean age of 14.8 years (SD: ±5.1 years).

**Table 1.** Cohort *RPE65* Variants

Patient Identifier	Gender	Age	Variant 1	Amino Acid Change 1	Variant 2	Amino Acid Change 2
MM_0349^*^	M	24	c.130C>T	p.Arg44Ter	c.1543C>T	p.Arg515
MM_0262^*^	F	21	c.118G>A	p.Gly40Ser	c.955G>A	p.Glu319Lys
MM_0350^*^	M	21	c.130C>T	p.Arg44Ter	c.1543C>T	p.Arg515
MM_0289^*^	F	20	c.989G>A	p.Cys330Tyr	c.1443_1445delAGA	p.Glu481del
MM_0313^*^	M	20	c.1398C>G	p.Tyr466Ter	c.1464T>A	p.Ser488Arg
MM_0304^*^	F	19	c.11+5G>A	Splice site alteration	c1341_1342dupCT	p.Cys448SerfsTer4
MM_0255^*^	M	19	c.1451G>A	p.Gly484Asp	c.1451G>A	p.Gly484Asp
MM_0220^*^	F	18	c.1078C>A	p.Pro363Thr	c.1078C>A	p.Pro363Thr
MM_0229^*^	M	18	c.11+5G>A	Splice site alteration	c.1102T>C	p.Tyr368His
MM_0309^*^	M	18	c.859G>T	p.Val287Phe	c.859G>T	p.Val287Phe
MM_0340^*^	M	17	c.370C>T	p.Arg124Ter	c.952T>A	p.Tyr318Asn
MM_0252^*^	M	16	c.11+5G>A	Splice site alteration	c.245G>A	p.Arg82Lys
MM_0264^*^	F	16	c.272G>A	p.Arg91Gln	c.1306G>A	p.Gly436Arg
MM_0278	M	16	c.952T>A	p.Tyr318Asn	c.852delC	p.Met285TrpfsTer40
MM_0277^*^	F	14	c.271C>T	p.Arg91Trp	c.271C>T	p.Arg91Trp
MM_0231^*^	M	14	c.1451G>A	p.Gly484Asp	c.1451G>A	p.Gly484Asp
MM_0234^*^	M	12	c.271C>T	p.Arg91Trp	c.1102T>C	p.Tyr368His
MM_0283^*^	F	11	c.353G>A	p.Arg118Lys	c.353g>A	p.Arg118Lys
MM_0392^*^	F	11	c.47T>C	p.Phe16Ser	c.1292A>G	p.Tyr431Cys
MM_0419	M	11	c.292_311del	p.Ile98HisfsTer26	c.1388C>A	pPro463His
MM_0423	F	11	c.370C>T	p.Arg124Ter	*RPE65* gene deletion encompassing at least exons 1 - 14	
MM_0292^*^	F	10	c.74C>T	p.Pro25Leu	c.11+5G>A	Splice site alteration
MM_0256	F	9	c.131G>A	p.Arg44Gln	c.1024T>C	p.Tyr342His
MM_0434	F	9	c.95-2A>T	Splice site alteration	c.311G>T	p.Gly104Val
MM_0368	F	5	c.304G>T	p.Glu102Ter	304G>T	p.Glu102Ter
MM_0413	M	5	c.331C>T	p.Pro111Ser	c.1451G>A	p.Gly484Asp

Shown are the age (years), variants, and associated amino acid changes in our cohort (*n* = 26).

* Indicates subjects previously described by authors.^13^

### Interocular Symmetry

**Figure 2.**
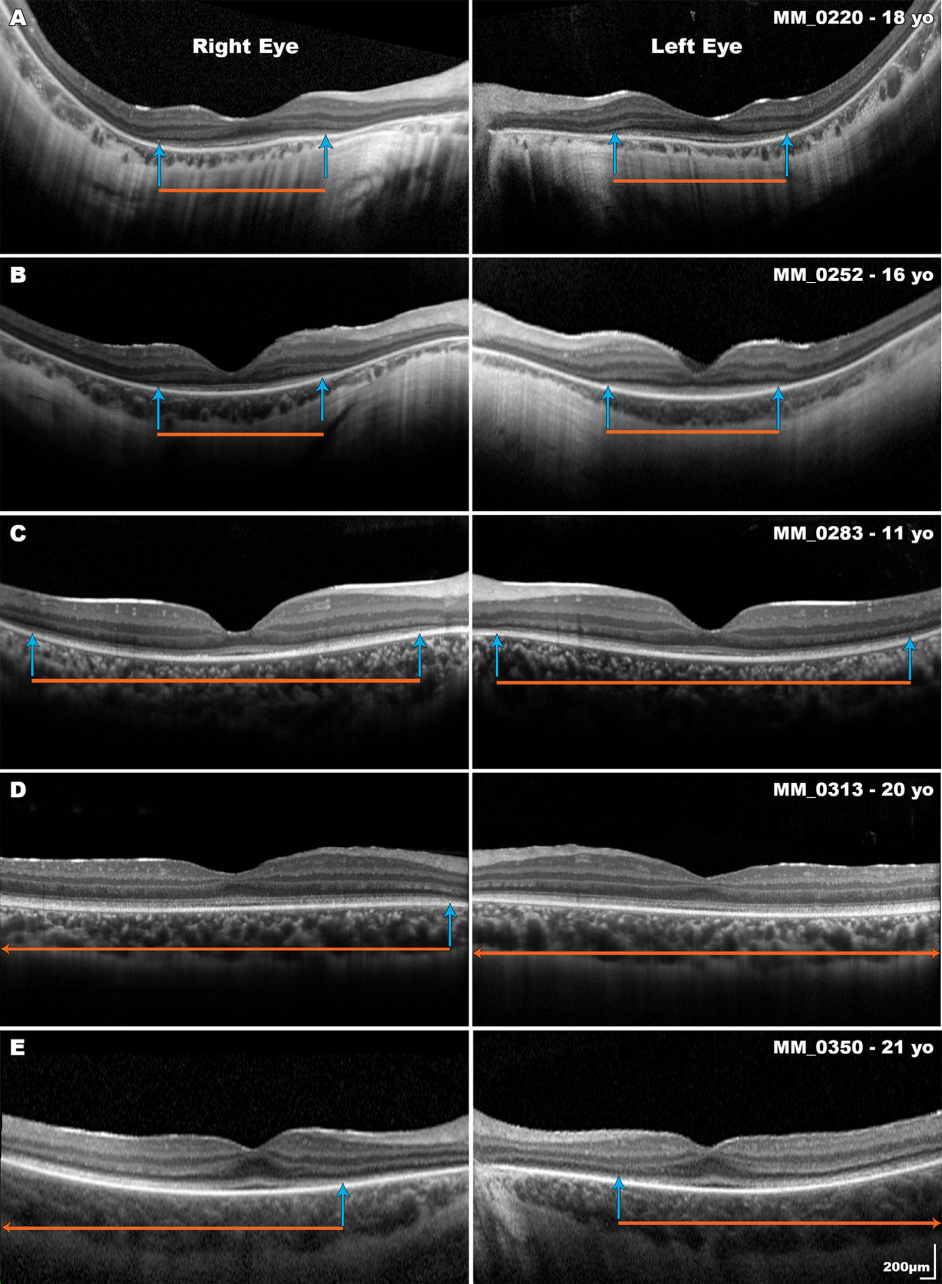
Disease symmetry in subjects with *RPE65*-associated RD. Interocular symmetry was observed in all five subjects. The orange bars mark the width of the ellipsoid zone and the blue arrows indicate the borders of where it is lost. Two of the five subjects have foveal hypoplasia and two subjects have an ellipsoid zone extending beyond the width of the scan (D and E). All scans are to the same scale.

Interocular symmetry was investigated for both baseline measurements and rates of change for each of the four metrics described. Investigation of normality identified that baseline right eye ONLT (*P* = 0.01, skewness = −1.45), baseline right eye EZW (*P* = 0.02), baseline right eye EZA (*P* = 0.02), and FT rate of change (*P* = 0.03, skewness = −1.17) data deviated from a normal distribution, whereas all others did not. Subsequently, no statistically significant differences (*P* < 0.05) were identified in baseline measurements or rates of change between left and right eyes, suggesting high interocular symmetry both at baseline and in terms of progressive change. Figure 2 demonstrates representative examples of interocular symmetry.

#### Cross-Sectional Analysis of Retinal Structure

FT and ONLT for both eyes of all 26 subjects were measured. EZW was measured for 35 of 52 eyes (67%; from 18 subjects). For the remaining 17 eyes, the EZW could not be measured as the EZ extended beyond the 30° scan (Figs. 2D, 2E). EZA was calculated for 19 of the 52 eyes (37%; from 10 subjects). The EZA could not be calculated for 24 eyes (46%) as the borders of the EZ extended beyond the 20° square volume scan, and for 9 eyes as the subjects’ nystagmus precluded adequate quality OCT volume scans.

### SD-OCT Baseline Results and Analysis of Agreement

**Figure 3.**
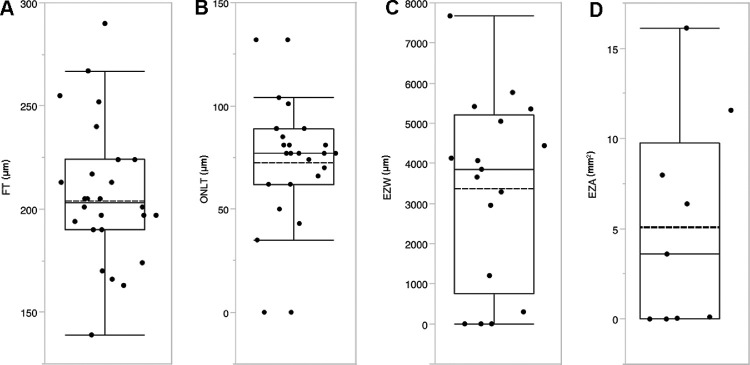
Combined stacked scatterplots and box plots for SD-OCT measurements. Shown are stacked scatterplots and box plots for (**A**) foveal thickness (FT, *n* = 26 eyes), (**B**) outer nuclear layer thickness (ONLT, *n* = 26 eyes), (**C**) ellipsoid zone width (EZW, *n* = 17 eyes), and (**D**) ellipsoid zone area (EZA, *n* = 9 eyes) for the left eyes of all patients, where measurable. Box plots with box spanning interquartile range with maximum and minimum values (excluding outliers) are provided. Solid and dashed lines represent median and mean, respectively.

Figure 3 shows combined stacked scatter plots and box plots for FT, ONLT, EZW, and EZA for the left eyes of all subjects. Mean ± SD FT and mean ± SD ONLT were 207 ± 34 µm and 73 ± 31 µm, respectively. Mean ± SD EZW was 3,362 ± 2,320 µm (*n* = 17 eyes) and mean ± SD EZA was 5.09 ± 5.9 mm^2^ (*n* = 9 eyes). Notably, one subject (MM_0340, 17 years old) had no measurable ONLT, EZW, or EZA; another subject (MM_0278, 16 years old) had no measurable ONLT or EZW on the line scan and a very small EZA (0.05 mm^2^), whereas a third subject (MM_0309, 18 years old) had an ONLT of 62 µm but no measurable EZW or EZA. This highlighted that subjects can ultimately completely lose OCT derived measures of outer retinal structure even at a relatively young age. Finally, a fourth subject (MM_0255, aged 19 years old) demonstrated very minimal residual outer retinal structure (ONLT, 66 µm; EZW, 299 µm; and EZA, 0.11 mm^2^).

**Figure 4.**
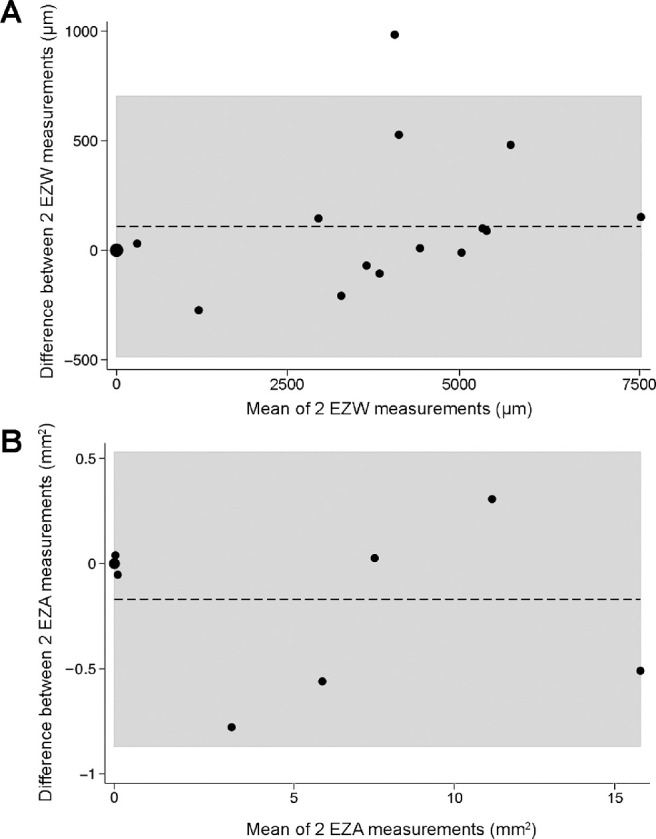
Agreement of SD-OCT ellipsoid zone measurements. (**A**) Bland-Altman plot of ellipsoid zone width (EZW; µm) demonstrating the mean difference (dashed line, 108.9 µm) and upper and lower limits of agreement (shaded area, −485.82 µm to 703.69 µm). (**B**) Bland-Altman plot of ellipsoid zone area (EZA; mm^2^) demonstrating the mean difference (dashed line, −0.17 mm^2^) and upper and lower limits of agreement (shaded area, −0.87 mm^2^ to 0.53 mm^2^).

Analysis of agreement is shown in Figure 4. The relatively small mean differences (EZW, 108.9 µm and EZA, −0.17 mm^2^), compared to the respective average baseline measurements, demonstrate overall good agreement in intra-observer measurements.

Foveal hypoplasia (Fig. 2: MM_0220 and MM_0350) was noted in 13 subjects (50%) and in all cases was bilateral. An independent *t*-test identified that subjects with foveal hypoplasia had a statistically significantly lower BCVA (1.07 ± 0.12 LogMAR) and, therefore, worse visual acuity compared to subjects without foveal hypoplasia (0.73 ± 0.08 LogMAR), t(24) = −2.474, *P* = 0.039.

### Association of SD-OCT With Retinal Function and Age

Correlation among all four SD-OCT metrics and BCVA, central 30° retinal sensitivity (V_30_) and age were investigated. This identified that only EZW had a statistically significant (*P* < 0.05) correlation with BCVA (r = −0.52, *P* = 0.03) and V_30_ (r = 0.57, *P* = 0.02). This suggests that patients with wider EZW and thereby greater preservation of retinal structure have better BCVA and larger volumes of retinal sensitivity. There was no correlation between age and any OCT metrics.

#### Longitudinal Analysis of Retinal Structure

Longitudinal data was available from 22 eyes of 14 subjects. FT and ONLT were calculated for all eyes, whereas EZW and EZA were calculated for 18 and 9 eyes, respectively.

Table 2 shows the rate of change of FT (µm/year), ONLT (µm/year), EZW (µm/year), and EZA (mm^2^/year) for all available eyes (*n* = 22). Follow-up ranged from 8 to 32 months, with a mean follow-up of 18 months (SD ± 6.7 months). The average FT, ONLT, EZW, and EZA rates of change were −1.75 µm/year, −1.43 µm/year, −330.63 µm/year, and −1.71 mm^2^/year, respectively. These rates of change account for 0.8%/year, 2.0%/year, 9.8%/year, and 23.0%/year of the respective average of FT, ONLT, EZW, and EZA described above.

**Table 2.** SD-OCT Rate of Change

Patient	Age at		Follow up				
Identifier	Baseline	Eye	(Months)	FT	ONLT	EZW	EZA
MM_0262	21	Right	19	4.89	0	−86.44	x
MM_0350	21	Right	16	5.8	5.81	x	x
		Left	9	−10.32	10.32	x	x
MM_0289	20	Right	20	11.61	9.29	−492.67	−1.71
MM_0304	19	Right	22	−4.22	−4.22	−268.83	−0.88
MM_0255	19	Left	28	−14.93	−6.63	−128.14	−0.07
MM_0220	18	Right	32	1.45	2.9	−433.78	−1.08
		Left	12	0	3.87	−11.04	−0.05
MM_0229	18	Right	17	2.73	−27.32	−241.72	−
		Left		−24.59	−2.73	−494.88	−
MM_0340	17	Left	18	10.32	0	0	0
MM_0252	16	Right	29	−22.42	−4.8	−428.61	−1.58
		Left	11	0	0	−368.71	−2.84
MM_0277	14	Right	12	−30.96	0	0	0
		Left		−7.74	−7.74	−140.3	−
MM_0231	14	Right	11	0	−21.11	−325.9	x
MM_0234	12	Right	25	5.57	−1.86	x	x
		Left		0	3.72	x	x
MM_0283	11	Right	20	2.32	2.32	−587.22	x
		Left		4.64	0	−784.31	x
MM_0292	10	Right	8	17.42	0	−85.48	x
		Left	14	9.95	6.63	67.94	x
**Average**	**15.9**	**N/A**	**18**	**−1.75**	**−1.43**	**−300.63**	**−1.17**
**SD**	**3.7**	**N/A**	**6.7**	**12.29**	**8.77**	**231.21**	**0.99**
**Range**	**10-21**	**N/A**	**8-32**	**−30.96** **to** **17.42**	**−27.32** **to** **10.32**	**−784.31** **to** **67.96**	**−2.84** **to** **−0.05**

Shown are the age (years), eye, and number of month's follow-up and the rate of change of the four metrics Measurements which were not possible due to the EZ extending beyond the scan width or nystagmus, have been identified with an “x” and “-,” respectively. Furthermore, the right eye of MM_0277 and the left eye of MM_0340 did not have a measurable ellipsoid zone at baseline, nor at follow-up, and so were not included in calculation of average and SD rate of change values for EZW and EZA. (Total change in SD-OCT derived metrics are available in Supplementary Table S1.)

**Figure 5.**
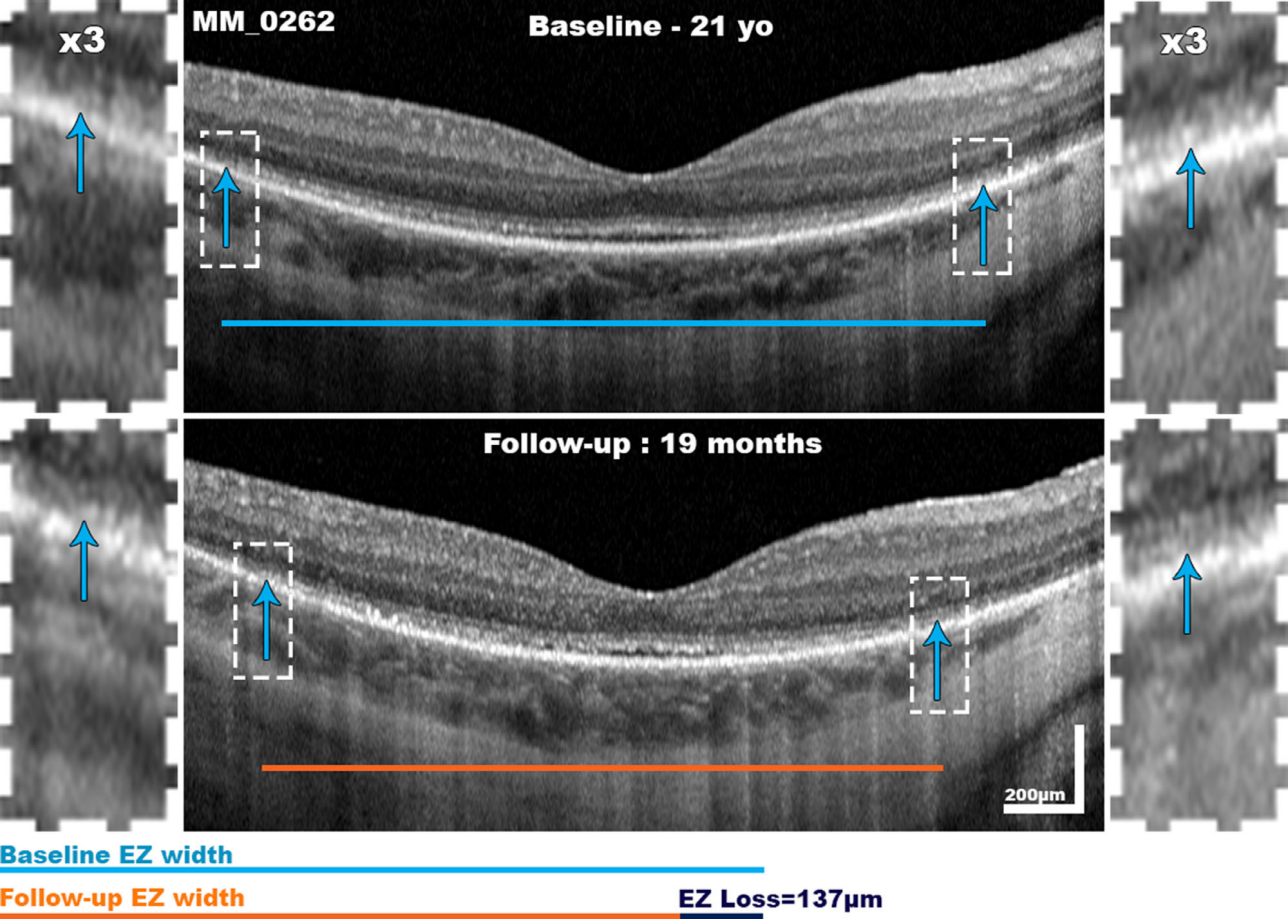
Ellipsoid zone width (EZW) progression. Demonstrated is an example of EZW progression in a 21-year-old subject (MM_0262) over 19 months.

Baseline values and final follow-up values were compared in the left eyes of subjects with a paired *t*-test. Interestingly, EZW values seemed smaller at follow-up (2615 ± 722µm) compared to baseline (2955 ± 783µm), however this did not quite reach statistical significance, t(7) = 2.071, *P* = 0.08. Figure 5 demonstrates progression of EZW in one subject. Similarly, no statistically significant difference was found between baseline and final follow-up measurements in the other three metrics of FT (*P* = 0.38), ONLT (*P* = 1.00), and EZA (*P* = 0.41).

Furthermore, on investigating the correlation of baseline measurements with rate of change, we identified that baseline FT had a strong, statistically significant, negative correlation with rate of change (r = −0.83, *P* < 0.01), suggesting that patients with thinner foveae progress slower; and it is these subjects in whom there seems to be some “increase” in FT. There was no correlation among ONLT, EZW, EZA, and baseline values, nor between rate of change in our cohort.

Furthermore, it is notable that seven eyes were identified as having foveal thinning (range, 8–54 µm). In comparison, 11 eyes were identified as having a greater FT at their follow-up visit compared to baseline (range, 4–19 µm). Similarly, eight eyes were identified as having ONL thinning (range, 3–38 µm). In comparison, eight eyes were identified as having greater ONLT at their follow-up visit compared to baseline (range, 4–8 µm). Subsequently, a relationship was identified between the presence of increased FT over time and the presence of foveal hypoplasia (*P* = 0.04).

### Fundus Autofluorescence

**Figure 6.**
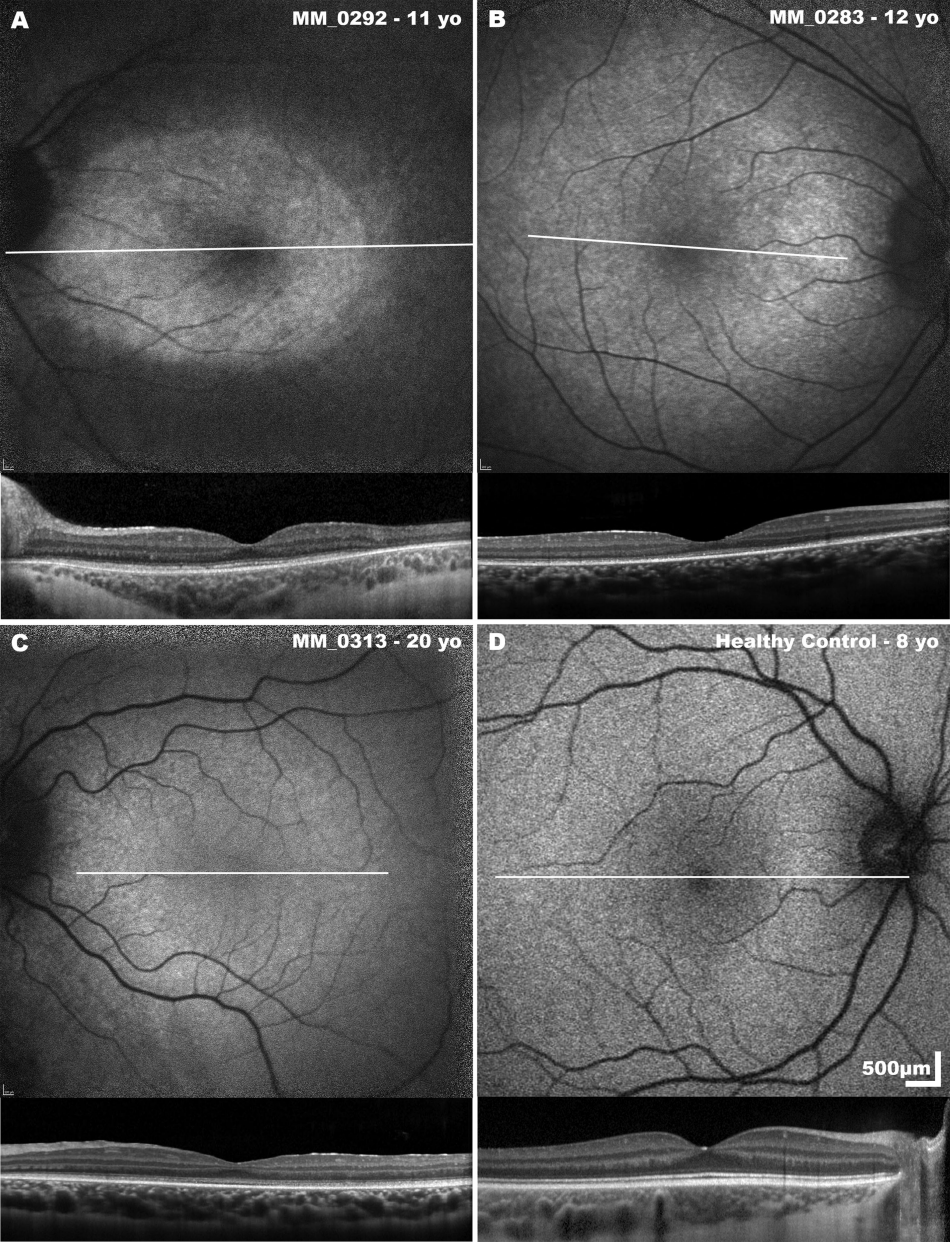
Examples of fundus autofluorescence phenotypes and associated SD-OCT scans seen in *RPE65*-RD. (**A–C**) The demonstrated increased central autofluorescence seen in some subjects with *RPE65*-RD, (**D**) demonstrates findings in a healthy control for comparison. White lines indicate the position and extent of the SD-OCT B-scans.

Twelve subjects (46%) demonstrated the presence of autofluorescence (Figs. 6A–6C) at the posterior pole, whereas 14 subjects (54%) demonstrated no autofluorescence. Interestingly, 8 of the 10 subjects demonstrated a ring of increased autofluorescence at the macula with foveal hypo-autofluorescence (attributed to macular pigment), on occasion extending beyond the retinal vascular arcades, with the background signal otherwise markedly reduced (Fig. 6). Furthermore, 2 of these 10 subjects showed increased autofluorescence at the fovea and perifovea with associated flecks of increased autofluorescence more peripherally. Furthermore, age did not correlate with presence of increased autofluorescence (*P* = 0.632).

Thirty-one eyes from 16 subjects were monitored using FAF longitudinally, demonstrating no change in qualitative binary FAF grading over time. Follow-up ranged from 3 to 29 months, with a mean follow-up of 15.7 months (SD ± 9.0 months.)

### Discussion

In this study, we have investigated retinal structure in the largest prospective cohort to date of subjects with *RPE65*-RD, using both SD-OCT and FAF, using novel metrics (EZW and EZA), exploring their agreement, and moreover have undertaken the first prospective assessment of retinal degeneration over time. This is especially relevant given the approved gene therapy, other on-going gene therapy trials (NCT02781480), and also that all previous *RPE65*-RD gene therapy trials noted retinal thinning following subretinal injection, which suggested continued retinal degeneration despite intervention.^42^^–^^45^

Studies to date have focused on retinal thickness and ONL thickness, with a need for a greater degree of interrogation of retinal integrity in *RPE65*-RD. Average (±SD) foveal thickness in a normative cohort (*n* = 50, age range, 20–69 years old) using the same instrument used in our study was 225.1 (±17.1) µm,^46^ which overlaps with the range of values in our cohort, suggesting no measurable change in foveal thickness in our subjects as compared to unaffected individuals. Notably, only 7 of the 22 eyes in our cohort demonstrated any foveal thinning during the follow-up period (18.0 ± 6.7 months).^47^ Similarly, mean (±SD) ONLT measurements in our cohort overlapped, to a lesser extent, with results from a normative cohort using a comparable methodology (*n* = 97, mean age ± SD, 29 ± 8 years), where a mean (±SD) ONLT of 105 (±12.2) µm was described.^48^ Interestingly, other studies have suggested ONL thinning, noted on TD-OCT, more marked in a smaller, older cohort.^19^^,^^20^ In a similar aged cohort, more minimal ONL thinning is noted, which is comparable to the findings presented herein, especially when taking into account the poorer (approximately 8 µm) resolution of TD-OCT.^19^^,^^20^

Similar to this study, other groups have not identified a progressive loss of retinal structure using FT and ONLT measurements alone.^22^ In contrast herein, EZW and EZA demonstrated that it is possible to quantifiably monitor photoreceptor loss, as all subjects (except in one eye of one subject; MM_0292) with residual EZ showed a slow progressive loss of EZW and EZA with longitudinal follow-up (Table 2). Furthermore, as shown in Figure 4, EZW and EZA both demonstrated a good level of agreement when measured twice, with small mean differences compared to their mean values, indicating that the measurement process itself is largely robust. Using a paired *t*-test, we demonstrated a trend toward worse EZW in patients at follow-up, but this did not reach statistical significance (*P* = 0.08). We suggest this to be because of the relatively slow rate of progression seen in this condition, coupled with a relatively short follow-up timeframe. Moreover, this also suggests that more sensitive advanced structural measurements, such as cellular imaging with nonconfocal adaptive optics imaging, may be better able to detect retinal degeneration in a more timely fashion.^49^

In investigating the relationship between baseline measurements and rate of change, we identified that subjects with thin FT measurements seemed to progress slower; which was not reflected in the similar ONLT, EZW, and EZA analysis. This, on the one hand, could suggest that residual retinal structure derived from other retinal layers may be more robust in end-stage *RPE65*-RD. However, FT thickening was noted to be more common in subjects with foveal hypoplasia (*P* = 0.04) suggesting more variability in FT measurements in subjects with foveal hypoplasia. As such, we suggest it may be challenging to use FT measurements to monitor retinal degeneration in *RPE65*-RD.

Of note, the 79.26 µm increase in EZW seen in the left eye of MM_0292 with follow-up (Supplementary Table S1) compares favorably to the 108.9 µm mean difference between two measurements (Fig. 4), suggesting this is within test-retest variability of this assessment.

Notably, of all four SD-OCT measures, only EZW demonstrated a statistically significant correlation with BCVA and retinal sensitivity of the central 30 degrees, suggesting greater structure-function correlation in *RPE65*-RD with EZW. Our study also identified a poor correlation of all SD-OCT metrics with age. We suggest this highlights the structural variability across different ages in *RPE65*-RD and the need to be aware that even relatively young children can have substantial loss of retinal structure.

Based on our results, we suggest EZW to be a better metric for quantifying retinal degeneration, compared to the other three investigated herein. First, as both EZW and EZA demonstrated longitudinal loss in outer retinal structure in all but one eye of one subject, in comparison to FT and ONLT, which demonstrated an apparent increase in measurement in multiple eyes (FT, *n* = 11 eyes and ONLT, *n* = 8 eyes); bearing in mind a ±1.8 µm estimated SD of repeatability of the Heidelberg Spectralis OCT2 (Heidelberg Engineering, Heidelberg, Germany).^50^ Second, in our study, EZW and EZA demonstrated an overall good level of agreement. Finally, because a greater number of subjects had a measurable EZW, compared to EZA, a greater structure-function correlation was also apparent with EZW.

We also demonstrated the presence of foveal hypoplasia in 50% of subjects, suggesting a possible role for RPE65 and/or a functioning visual cycle, for normal macular development. Interestingly, subjects with foveal hypoplasia also had a significantly worse BCVA (*P* = 0.039). This may suggest that these patients may have less potential to benefit from intervention compared to patients without foveal hypoplasia.

In contrast to a previous study,^30^ 46% of our subjects demonstrated the presence of central autofluorescence, with some demonstrating a ring of increased autofluorescence at the macula with foveal hypo-autofluorescence. This could suggest an abnormal accumulation of lipofuscin in the RPE, as a result of increased outer segment degeneration.^51^ Interestingly, a similar pattern of central hyperautofluorescent rings have been identified in subjects with hypomorphic *RPE65*-RD.^31^ Of note, no change in binary grading (presence or absence) was identified on longitudinal follow-up and there does not seem to be a correlation with the presence of autofluorescence and age, similar to the assessment of retinal structure with SD-OCT.

There are, however, several limitations to our study. First, a longer and more uniform follow-up of both eyes and a larger number of subjects would enable us to better examine longitudinal loss of retinal structure with SD-OCT. Future studies with multiple OCT assessments over a longer time frame will also provide insight into possible nonlinear longitudinal loss of retinal structure. In addition, our SD-OCT protocol did not include directional OCT; whereas the majority of ONLT will be accounted for by the ONL (containing solely photoreceptor nuclei), a small proportion of the thickness will be due to the Henle fiber layer (containing photoreceptor axons and Muller cell processes), which can be accounted for by directional OCT.^52^ Additionally, EZW measurement and, more-so, EZA measurements, are limited by the acquisition width and square area of 30° and 20°, respectively, meaning that only data from 67% and 37% could be quantified by these metrics, respectively. All patients excluded from EZW analysis and 46% of patients excluded from EZA analysis by definition had better retinal structure and, therefore, possibly a milder or less advanced phenotype. Widefield OCT may help to address this imaging limitation. Furthermore, FAF analysis in our study was limited to qualitative assessment alone, which (even when binary, as performed herein) may be challenging. Future longitudinal evaluation with *quantitative* FAF will be valuable. However, quantification of poorly demarcated areas of FAF in other inherited retinal disease can be difficult.^53^

In conclusion, our detailed investigation of retinal integrity will be important in identifying and monitoring subjects with *RPE65*-RD in both treatment and clinical trial settings – both in terms of safety, but moreover, in terms of addressing one of the primary clinical objectives of slowing or halting otherwise inexorable retinal degeneration.

## Supplementary Material

Supplement 1
