# Supplementary material for: Retinal Structure in RPE65-Associated Retinal Dystrophy
Source: Invest Ophthalmol Vis Sci. 2020 Apr 29;61(4):47. doi: 10.1167/iovs.61.4.47 (PMC7401957; doi:10.1167/iovs.61.4.47)
Supplement: Supplement 1 [file iovs-61-4-47_s001.pdf]

Retinal structure in *RPE65*-associated retinal dystrophy – Supplementary Material

Supplementary Table

Patient Identifier	Age at baseline	Eye	Follow up (months)	FT	ONLT	EZW	EZA
MM_0262	21	Right	19	7.74	0.00	-136.86	x
MM_0350	21	Right	16	7.74	7.74	x	x
		Left	9	-7.74	7.74	x	x
MM_0289	20	Right	20	19.35	15.48	-821.11	-2.85
MM_0304	19	Right	22	-7.74	-7.74	-492.85	-1.62
MM_0255	19	Left	28	-34.83	-15.48	-298.99	-0.16
MM_0220	18	Right	32	3.87	7.74	-1156.76	-2.88
		Left	12	0.00	3.87	-11.04	-0.05
MM_0229	18	Right	17	3.87	-38.70	-342.44	-
		Left		-34.83	-3.87	-701.07	-
MM_0340	17	Left	18	15.48	0.00	0.00	0.00
MM_0252	16	Right	29	-54.18	-11.61	-1035.82	-3.81
		Left	11	0.00	0.00	-337.99	-2.61
MM_0277	14	Right	12	-30.96	0.00	0.00	0.00
		Left		-7.74	-7.74	-140.30	-
MM_0231	14	Right	11	0.00	-19.35	-298.74	x
MM_0234	12	Right	25	11.61	-3.87	x	x
		Left		0.00	7.74	x	x
MM_0283	11	Right	20	3.87	3.87	-978.69	x
		Left		7.74	0.00	-1307.18	x
MM_0292	10	Right	8	11.61	0.00	-56.98	x
		Left	14	11.61	7.74	79.26	x

Supplementary Table S1: SD-OCT change. Shown are the age (years), eye, number of months follow up, and the total change of the four metrics: FT; foveal thickness (µm), ONLT; outer nuclear layer thickness (µm), EZW; ellipsoid zone width (µm), and EZA; ellipsoid zone area (mm^2). Measurements which were not possible due to the EZ extending beyond the scan width or nystagmus, have been identified with an 'x' and '-', respectively. Furthermore,

the right eye of MM_0277 and the left eye of MM_0340 did not have a measurable ellipsoid zone at baseline, nor at follow up.
